# Adequacy of public maternal care services in Brazil

**DOI:** 10.1186/s12978-016-0229-6

**Published:** 2016-10-17

**Authors:** Sonia Duarte de Azevedo Bittencourt, Rosa Maria Soares Madeira Domingues, Lenice Gnocchi da Costa Reis, Márcia Melo Ramos, Maria do Carmo Leal

**Affiliations:** 1 Escola Nacional de Saúde Pública Sérgio Arouca, Fundação Oswaldo Cruz, Rio de Janeiro, Brazil; 2 Instituto Nacional de Infectologia Evandro Chagas, Fundação Oswaldo Cruz, Rio de Janeiro, Brazil; 3 Subsecretaria de Vigilância, Fiscalização Sanitária e Controle de Zoonose, Secretaria Municipal de Saúde, Rio de Janeiro, Brazil

**Keywords:** Maternal health, Maternity hospitals, Structure of services, Quality of health care

## Abstract

**Background:**

In Brazil, hospital childbirth care is available to all, but differences in access and quality of care result in inequalities of maternal health. The objective of this study is to assess the infrastructure and staffing of publicly financed labor and birth care in Brazil and its adequacy according to clinical and obstetric conditions potentially associated with obstetric emergencies.

**Methods:**

Nationwide cross-sectional hospital-based study “Birth in Brazil: national survey into labor and birth” conducted in 2011–2012. Data from 209 hospitals classified as public (public funding and management) or mixed (public or private funding and private management) that generate estimates for 1148 Brazilian hospitals. Interview with hospital managers provided data for the structure adequacy assessment covering four domains: human resources, medications, equipment for women emergency care and support services. We conducted analysis of the structure adequacy rate according to type of hospital (public or mixed), availability of ICU and the woman obstetric risk using the *X* ^2^ test to detect differences in categorical variables with the level of statistical significance set at *p* <0.05.

**Results:**

Global rate of adequacy of 34.8 %: 42.2 % in public hospitals and 29.0 % in mixed hospitals (*p* < 0.001). Public and mixed hospitals with ICU had higher scores of adequacy than hospitals without ICU (73.3 % × 24.4 % public hospitals; 40.3 % × 10.6 % mixed hospitals). At a national level, 32.8 % of women with obstetric risk were cared for in hospitals without ICU and 29.5 % of women without risk were cared for in hospitals with ICU. Inequalities were observed with the North, Northeast and non-capital regions having the lower rates of hospitals with ICU.

**Conclusions:**

The majority of maternity wards across the country have a low rate of adequacy that can affect the quality of labor and birth care. This holds true for women at high obstetric risk, who suffer the possibility of having their care compromised by failures of hospital infrastructure, and for women at low obstetric risk, who may not receive the appropriate care to support the natural evolution of their labor when in a technological hospital environment.

## Background

The majority of maternal deaths could be avoided with timely implementation of appropriate measures of recognized efficacy. Obstacles to these goals are delays in seeking care, in reaching the correct care setting, in awaiting provider attention, and, of course, in receiving the correct workup and management [1].

Improvement in maternal and neonatal outcomes depends upon multifactorial intervention strategies tailored to the specific context [2], such as to ensure general access to effective care. It is not enough to offer access without a mechanism to support appropriate care, because the provision of effective management to a small portion of the population can exacerbate inequalities within a health care system. As such, weaknesses with respect to access and quality must be identified and confronted to properly buttress these strategies.

Coordination of a network of obstetric services plays a fundamental role in maternal health for a large country like Brazil and in the current global scenario of perennially squeezed budgets as health care technology continues to drive rising costs. It is not only a matter of making health services locally available but also of endowing these services with the applicable infrastructure and integrating them into a network. This depends upon communicative and cooperative management that enables interaction among diverse entities.

In Brazil, providing specialized maternity services to women at childbirth is a legal requirement since 2007 [3]. Nevertheless, guaranteeing hospital admission for pregnant women continues to be a challenge, even in cases of clinical or obstetric risk during prenatal care. The phenomenon of pregnant women traveling to multiple access points in search of appropriate care remains commonplace in Brazil [4]. This situation is most dramatic in cases of obstetric emergencies that cry out for an agile referral system. Poor communication between local services and those coordinating bodies responsible for effecting transfer to appropriate care settings is a key factor associated with delays in adequate delivery of care, specifically in the arena of maternal and child health [5].

Brazil’s persistently elevated levels of maternal and neonatal morbidity and mortality underscore ongoing problems in this sector. A previous study using data from the “Birth in Brazil” study has shown low rates of adequacy of maternal care services for neonatal care in Brazil [6]. Other countries, like Lebanon [7], India [8] and the Dominican Republic [9] have a similar situation. Providing high-quality care to pregnant women, especially those with potentially fatal obstetric complications, could contribute greatly to a reduction in maternal morbidity and mortality [9, 10].

The Brazilian health system mixes public and private financing [11]. The private sector is composed of mixed hospitals - which have beds paid through private funds or contracted by the government - and private hospitals, covered by private health insurance or out of pocket payments. Public and private services provide childbirth care in Brazil and nearly 80 % of all women have public funding of childbirth care offered by public or mixed hospitals [12]. Previous studies have shown that maternal and neonatal outcomes differ between the different types of hospital [13, 14] and geographical locations [15].

The objective of this study is to assess the adequacy of the infrastructure and staffing of publicly financed labor and birth care services in Brazil according to Brazilian macro region, type of service and clinical and obstetric conditions potentially associated with obstetric emergencies.

## Methods

This study used data from the “Birth in Brazil” study, conducted between February 2011 and October 2012. The national hospital-based investigation of peripartum women and their newborns also included data regarding the organization and facilities at their sites of care. The sampling process used three stages. First, we stratified hospitals with 500 or more annual births by macro region (North, Northeast, Southeast, South, and Central-West), locality (capital or non-capital), and type of service (public, private, or mixed). In the second stage, we selected the number of days needed to interview 90 puerperal women at each hospital, with a minimum of 7 days, using an inverse sampling method. In the third stage, we interviewed 90 women at each selected hospital. Data from the Birth in Brazil study can generate estimates for 1402 hospitals based on the 266 hospitals that we visited for data collection. More information regarding the sampling process is available in a previous publication [16].

We carried out face-to-face interviews with postpartum women during their hospital stays. We extracted data from the medical records for both mother and newborn and photographed prenatal care cards for further data collection. Finally, we obtained information about the facility structure through an interview with the hospital director. We used a structured questionnaire containing information about the availability of human resources, medications, equipment and support services as well as the use of clinical guidelines and the use of process and outcome indicators. More details on data collection instruments are available in Leal et al [17] and Bittencourt et al [18].

There were 209 participating hospitals classified as either public (public funding and management) or mixed (public or private funding and private management) that generate estimates for 1148 Brazilian hospitals. With respect to public hospitals, all interviewed women were included in the analysis; for mixed hospitals, only interviews with women with public funded births were included. Therefore, we excluded all interviews with women with private insurance paid births. Included interviews made up 88.3 % of all interviews conducted in mixed-funding hospitals.

Based on interviews with hospital directors, we classified each hospital investigated according to whether it had an adult Intensive Care Unit (ICU). We investigated four further hospital facility or organization domains: a) human resources; b) medications; c) equipment for emergency care of pregnant women; and d) support services. Criteria for human resources were as follows: presence of a nurse or physician who can risk-stratify each presenting pregnant woman, and at least one obstetrician and/or anesthesiologist working by shifts at the facilities, without interruption. We checked the availability of eight medications classes, in accordance with Brazilian regulations: antihypertensive, anxiolytics or hypnotics, corticosteroids, oxytocic, tocolytic, antihemorrhagics, magnesium sulfate, and anti-D immunoglobulin for Rh-negative women. For the domain of equipment for the emergency care of pregnant women, we investigated the presence of the following items: laryngoscopes and orotracheal tubes, mechanical ventilators, and manual resuscitators. We checked the availability of the following support services: blood bank or transfusion unit, clinical pathology laboratory, and ambulance transport. For facilities with ICU, that provide care to high-risk women, we assessed four further categories within human resources: 1) presence of “medical director of obstetrics”; 2) presence of “nursing director of obstetrics”; 3) medical director with specialized training in obstetrics; 4) nursing director with specialized training in obstetrics.

We calculated the adequacy score for each dimension using the percentage of positive answers relative to the total number of questions pertaining to each domain. We followed the same method for each group of domains. We assigned the resulting percentages to the following evaluations: adequate (between 90 and 100 %); partially adequate (between 70 and 89 %), inadequate (below 70 %). We gave equal weight to all assessed domains, with the maximum total varying between 17 and 21 points (for hospitals without and with ICU, respectively).

For each of the studied public and mixed-funding maternity hospitals, we classified enrolled women according to obstetric risk. Those considered at obstetric risk were those with at least one of the following complications: hypertensive syndromes (chronic hypertension, preeclampsia, eclampsia, HELLP syndrome), diabetes, placenta previa, placental abruption, and HIV or other diagnosis of infection at the time of hospital admission. We classified the remaining women as low obstetric risk. We based the categorization of risk on each woman’s medical record and on the prenatal care card, when available.

We analyzed data according to hospital type (public or mixed) and stratified by region (North/Northeast and Southeast/South/Central-West), locality (capital/non-capital), and presence of ICU. We examined the distribution of women classified as at obstetric risk by hospital adequacy score. We carried out comparison between categories using the chi-squared test, with significance set at *p* < 0.05.

We based the sampling weights for each hospital on the inverse of the probability of inclusion in the sample. To ensure that the total estimates were equal to the number of sampled hospitals, we used a calibration process for each stratum. We carried out all data analyses using IBM SPSS statistical software, Version 17.0 (IBM Corp., Armonk, NY, USA).

The research project was approved by the Ethics Committee for the National School of Public Health, Sérgio Arouca/Fiocruz (protocol number 92/2010).

## Results

We included 19,128 women in the analysis, corresponding to 80.1 % of the total sample for the Birth in Brazil study. Of this total, we classified 3664 women as at obstetric risk (19.2 %), with a greater prevalence in public hospitals located in capitals and in public and mixed hospitals located in the Southeast/South/Central-West regions (Table 1). It is noteworthy that the excluded women with private insurance paid births in mixed hospitals represented the group with the highest obstetric risk, relative to those women with public funding who were cared for in the same hospitals (22.9 % versus 17.5 %, *p* < 0.001). Without this exclusion, we estimated 18.1 % (95 % CI: 16.4–19.9 %) of women at obstetric risk in mixed hospitals. This figure is slightly higher, but not significantly different from the estimate when only women with public funding were included (17.5 %, 95 % CI: 15.7–19.5 %).

**Table 1:** Prevalence of obstetric risk and proportion of hospitals with Intensive Care Unit (ICU) according to type of hospital (public or mixed) and geographic location (Brazil, 2011–2012)

		Prevalence of obstetric risk % (n)	*p* value	Hospitals with Intensive Care Unit		*p* value
				No % (n)	Yes % (n)	
Public	Macro-Region					
	North/Northeast	18.6 (931)	<0.001	79.2 (221)	20.8 (58)	<0.001
	Southeast/South/Central-West	22.9 (1109)		43.7(100)	56.3 (129)	
	Locality					
	Non-Capital	16.6 (926)	<0.001	76.2 (112)	23.8 (35)	<0.001
	Capital	26.1 (1114)		31.0 (112)	69.0 (249)	
	Total	20.7 (2040)		63.2 (321)	36.8 (187)	
Mixed	Macro-Region					
	North/Northeast	13.1 (371)	<0.001	46.0 (58)	54.0 (68)	0.049
	Southeast/South/Central-West	19.4 (1253)		36.5 (188)	63.5 (327)	
	Locality					
	Non-Capital	17.3 (1120)	0.537	38.5 (33)	61.5 (52)	0.811
	Capital	17.8 (504)		37.6 (209)	62.4 (347)	
	Total	17.5 (1624)		38.4 (246)	61.6 (395)	
Brazil		19.2 (3664)		49.3 (567)	50.7 (582)	

Data from the 209 hospitals included in this analysis generated estimates for 1148 public and mixed hospitals that provide childbirth care in Brazil. Mixed hospitals had greater availability of ICU beds (61.6 %), compared with 36.8 % in the public setting (Table 1). The North/Northeast regions of the public system had the most unfavorable percentage, with only 20.8 % of hospitals equipped with ICU beds. The South, Southeast, and Central-West regions had the highest proportion of maternities with ICU beds in both public and mixed sectors. The availability of ICU beds in public hospitals located in capitals was almost three times higher the availability in non-capital cities while in mixed hospitals these values were nearly the same in capital and non-capital cities.

Table 2 presents the percentage of positive answers to each item assessed. Among hospitals with ICU, mixed hospitals were found to be the least likely to have physicians and/or nurses available to triage pregnant women, physicians and anesthesiologist available 24 h as well as a medical director with specialized training in obstetrics. In public facilities with ICU, the greatest observed deficit was in nursing directors with specialized training in obstetrics. Obstetrician and/or anesthesiologist working by shifts at the facilities were also less available in mixed hospitals without ICU. The availability of medications and of essential equipment to ensure maternal survival was similar between the two types of hospital with ICU. In hospitals without ICU, the greatest need in these categories was for both mechanical ventilators and antihemorrhagics and anti-D immunoglobulin for Rh-negative women in public hospitals. Public hospitals had more clinical pathology laboratory and ambulance transport in hospitals with or without ICU. We did not observe differences in the availability of blood bank or transfusion centers between public and mixed hospitals. Almost half of the hospitals without ICU did not have a blood bank or transfusion center.

**Table 2:** Proportion of adequacy items (availability of human resources, equipment for maternal emergency care, medications and supportive services) according to type of hospital and availability of Intensive Care Unit (ICU) (Brazil, 2011–2012)

Adequacy items	ICU available			ICU not available		
	Public % (n)	Mixed % (n)	*p* value*	Public % (n)	Mixed % (n)	*p* value*
Human resources						
Presence of a nurse or physician who risk-stratified each presenting pregnant woman	71.0 (132)	33.0 (130)	<0.001	42.8 (137)	51.8 (127)	0.033
Obstetrician 24 h	93.0 (174)	80.5 (318)	<0.001	85.3 (273)	40.8 (100)	<0.001
Anesthesiologist 24 h	81.7 (152)	46.6 (184)	<0.001	47.0 (151)	15.0 (37)	<0.001
Medical director of obstetrics with specialized training in obstetrics	96.2 (179)	88.9 (351)	0.006	-		
Nursing director of obstetrics with specialized training in obstetrics	52.9 (99)	52.3 (206)	0.818	-		
Equipment for the emergency care of pregnant women						
Laryngoscopes and orotracheal tubes,	100 (187)	99.5 (393)	0.330	87.5 (281)	95.5 (235)	0.001
Mechanical ventilators	88.7 (165)	89.4 (353)	0.812	66.0 (212)	91.0 (223)	<0.001
Manual resuscitators	100 (187)	99.5 (393)	0.330	95.0 (305)	100 (246)	< 0.001
Medications						
Antihypertensives	99.5 (185)	100 (395)	0.145	100 (320)	100 (246)	-
Anxiolytics/Hypnotics	94.7 (177)	99.0 (391)	< 0.001	96.0 (308)	89.8 (221)	0.004
Corticosteroids	100 (187)	99.2 (391)	0.232	97.5 (313)	98.0 (241)	0.717
Ocytocics	100.0 (187)	100 (395)	-	100 (320)	100 (246)	-
Tocolytics	97.3 (181)	96.7 (382)	0.696	100 (320)	100 (246)	-
Antihemorrhagics for women	86.6 (161)	87.1 (344)	0.860	76.9 (246)	95.9 (235)	<0.001
Magnesium sulfate	100.0 (187)	97.2 (384)	0.021	98.4 (315)	100 (246)	0.049
Anti-D immunoglobulin for Rh-negative women	95.2 (177)	94.4 (373)	0.715	76.9 (247)	92.3 (227)	<0.001
Support services						
Clinical Pathology Laboratory	98.9 (184)	82.8 (327)	< 0.001	87.9 (282)	67.1 (165)	< 0.001
Ambulance for transport of the women	97.8 (182)	89.1 (351)	<0.001	97.2 (312)	82.1 (202)	< 0.001
Blood bank or Transfusion unit	89.8 (167)	86.8 (343)	0.311	56.6 (181)	56.5 (139)	0.989

*Chi-square test

The global adequacy and the domains adequacy varied according to hospital type and complexity of the facilities (Table 3). Public hospitals had a higher global score and a higher score in the domains human resources and support services than mixed hospitals in both services with or without ICU. Mixed hospitals scored higher in medications in both types of hospital and in equipment in hospitals without ICU. Public hospitals with ICU had better scores in all domains when compared to public hospitals without ICU. Mixed hospitals with ICU had a higher global score than mixed hospitals without ICU mainly because of differences in the availability of human resources and support services (statistical analysis not shown in Table 3). At a national level, 34.8 % of hospitals were rated as adequate (42.2 % public, 29.0 % mixed, *p* < 0.001).

**Table 3:** Adequacy rates of human resources, equipment for the emergency care of pregnant women, medications and support services according to type of hospital (public or mixed) and availability of Intensive Care Unit (ICU) (Brazil, 2011–2012)

Adequacy items	ICU available			ICU not available		
	Public % (n)	Mixed % (n)	*p* value *	Public % (n)	Mixed % (n)	*p* value *
Human resources						
Adequate	22.5 (42)	13.2 (52)	<0.001	21.3 (68)	5.7 (14)	<0.001
Partially Adequate	68.4 (128)	49.1 (193)		39.7 (127)	21.2 (52)	
Inadequate	9.1 (17)	37.7 (148)		39.1(125)	73.1(179)	
Equipment for the emergency care of pregnant women						
Adequate	88.7 (165)	89.6 (353)	0.551	60.1 (193)	88.2 (217)	<0.001
Partially Adequate	11.3 (21)	9.9 (39)		33.3 (107)	9.8 (24)	
Inadequate	0.0 (0)	0.5 (2)		6.6 (21)	2.0 (5)	
Medications						
Adequate	78.0 (145)	80.6 (319)	<0.001	54.7 (175)	78.0 (191)	<0.001
Partially Adequate	22.0 (41)	16.2 (64)		42.8 (137)	22.0 (54)	
Inadequate	0.0 (0)	3.3 (13)		2.5 (8)	0.0 (0)	
Support services						
Adequate	88.7 (165)	67.6 (267)	<0.001	51.7 (166)	46.7 (114)	<0.001
Partially Adequate	9.1 (17)	25.6 (101)		38.0 (122)	20.9 (51)	
Inadequate	2.2 (4)	6.8 (27)		10.3 (33)	32.4 (59)	
Global adequacy evaluation						
Adequate	73.3 (137)	40.3 (159)	<0.001	24.4 (78)	10.6 (26)	<0.001
Partially Adequate	26.7 (50)	55.4 (218)		75.6 (242)	78.8 (193)	
Inadequate	0.0 (0)	4.4 (17)		0.0 (0)	10.6 (26)	

*Chi-square test

Table 4 presents the distribution of pregnant women classified as at obstetric risk, according to hospital type (public or mixed), geographic distribution and availability of ICU and adequacy score (no ICU; partially adequate or inadequate with ICU; adequate with ICU). In the public sector, 38.7 % of pregnant women considered at obstetric risk delivered in maternity hospitals without ICU. This value increased to 50.5 % in the North/Northeast regions and 55.1 % in the non-capital regions of Brazil. In mixed hospitals, about one-fourth of women at obstetric risk were cared for in hospitals with no ICU, irrespective of geographic location. At a national level, less than half of the women with obstetric risk were cared for in hospitals with ICU with adequate structure.

**Table 4:** Number and proportion of women with or without obstetric risk cared for in maternity services according to the availability of Intensive Care Unit (ICU), facility adequacy score, type of hospital and geographical location (Brazil, 2011–2012)

Type of hospital	Obstetric risk (n)		Intensive Care Unit (ICU)			*p* value*
			No ICU (%)	Inadequate/Partially Adequate (%)	Adequate (%)	
Public						
North/Northeast	No	(4066)	70.2	20.7	9.1	< 0.001
	Yes	(931)	50.5	28.4	21.2	
	Total	(4997)	66.5	22.1	11.4	
Southeast/South/Central-West	No	(3731)	39.5	8.4	52.1	< 0.001
	Yes	(1109)	28.9	5.7	65.4	
	Total	(4840)	37.1	7.8	55.1	
Capital	No	(3148)	41.1	19.3	39.6	< 0.001
	Yes	(1114)	25.1	17.7	57.3	
	Total	(4262)	36.9	18.8	44.2	
No Capital	No	(4649)	65.2	11.8	22.9	< 0.001
	Yes	(926)	55.1	14.1	30.8	
	Total	(5575)	63.6	12.2	24.3	
Total public	No	(7797)	55.5	14.8	29.7	< 0.001
	Yes	(2040)	38.7	16.0	45.2	
	Total	(9837)	52.0	15.1	32.9	
Mixed						
North/Northeast	No	(2464)	38.0	46.6	15.4	< 0.001
	Yes	(371)	26.1	33.1	40.9	
	Total	(2836)	36.5	44.8	18.8	
Southeast/South/Central-West	No	(5203)	31.0	33.0	36.0	< 0.001
	Yes	(1253)	25.2	32.6	42.2	
	Total	(6456)	29.9	33.9	37.2	
Capital	No	(2320)	29.5	45.2	25.4	< 0.001
	Yes	(504)	27.8	36.9	35.3	
	Total	(2824)	29.2	43.7	27.2	
No Capital	No	(5347)	34.9	33.9	31.2	< 0.001
	Yes	(1120)	24.4	30.8	44.8	
	Total	(6467)	33.1	33.4	33.5	
Total mixed	No	(7667)	33.3	37.3	29.4	< 0.001
	Yes	(1624)	25.4	32.7	41.9	
	Total	(9291)	31.9	36.5	31.6	
Brazil	No	(15464)	44.5	26.0	29.5	<0.001
	Yes	(3664)	32.8	23.4	43.8	
	Total	(19128)	42.2	25.5	32.3	

*Chi-square test

Table 4 also describes service utilization among women at low obstetric risk according to availability of ICU and adequacy rating. In the public system, admission to hospitals without ICU predominated in the North/Northeast regions (70.2 %). In the South, Southeast, and Central-West regions, we observed the reverse with 52.1 % of low-risk women cared for in adequate hospitals with ICU. We also observed differences among capital and non-capital cities, with higher proportions of low-risk women cared for in adequate hospitals with ICU in capital cities. In mixed hospitals, 37.3 % of low risk pregnancies were attended in partially adequate maternity wards with ICU, more than twice the value observed in public services. We also observed higher proportions of low risk women admitted to adequate mixed hospitals with ICU in the Southeast/South/Central-west regions. Overall, 55.5 % of Brazilian low-risk women were cared for in hospitals with ICU.

## Discussion

The “Birth in Brazil” study is the first nationwide hospital-based study that assessed the structure of public and private maternity services in Brazil. It used a sampling process that can generate estimates for the five macro-regions of the country, type of service (public, private and mixed) and service location (capital or non-capital), which are relevant to policy makers and those involved in the planning and delivery of childbirth care in Brazil.

However, the present analysis has some limitations. The Birth in Brazil study only examined hospitals that handle more than 500 deliveries per year, which is where more than 80 % of births occur nationally. Hospitals with lower birth volumes were not included and could represent a group with inferior care infrastructure, resulting in overestimation of the overall level of care.

We obtained data related to the structure of hospitals through interviews with hospital directors, not by direct observation on the part of the researchers, leading to the introduction of potentially significant biases, if hospital directors reported a more adequate structure, which could have led to an overestimation of adequacy.

Private hospitals and women who attended mixed hospitals, but who had their care paid for by private insurance, were not included in this study. Although these women cared for in mixed hospitals had a higher prevalence of obstetric risk, this most likely did not significantly alter the estimated risk in mixed hospitals owing to the low proportion (11.7 %) of private-funding cases in these settings.

Finally, we used equal weights for all items assessed and the items may have different importance in obstetric care. However, all items are essential to the quality of maternal care and we didn’t find published studies that could inform the weights to use.

The results of this study demonstrate a low rate of adequacy of public and mixed hospitals in Brazil: we rated only 34.8 % of the hospitals as adequate. Public hospitals presented a better score for human resources except for the presence of a nursing director with specialized training in obstetrics in hospitals with ICU, where only approximately half of both public and mixed hospitals met this criterion. While other studies have concluded that good outcomes in labor and delivery care often depend upon the number of qualified professionals available [19, 20] the present study only assessed the presence of physicians and nurses. When tasks are under the direct responsibility of these professionals, such as exams for admission to maternity wards, the absence of physicians and/or nurses can make the detection of high-risk obstetric conditions much more difficult. In the same way, the lack of an available obstetrician and/or anesthesiologist on a maternity ward [8] can lead to errors in the management of evolving labor, and consequently, cause delays between the decision to intervene with cesarean section and the actual procedure [21]. The disastrous effects of late care and insufficient professional attention on the needs of laboring women have been documented by various authors [5, 22].

Public hospitals also scored higher for support services, while mixed hospitals scored higher for medications. In hospitals without ICU, mixed hospitals also had a higher score for equipment. Overall, public hospitals with or without ICU scored higher than mixed hospitals. Specific characteristics of the private sector can explain some of these differences, such as the contract of support services, instead of having these services at the hospital. However, these differences can affect the quality of care. Ambulance services were least available in mixed hospitals without ICU that also had deficiencies in human resources, blood banking or transfusion units, and laboratory and clinical pathology facilities. These issues highlight inequalities in the distribution of staffing, materials and services indispensable to the proper support of labor and birth care.

Hospitals without Intensive Care Unit beds had lower global rates of adequacy: we rated only 24.4 % of public hospitals and 10.6 % of mixed hospitals without ICU as adequate. Public hospitals with an ICU scored better in all the assessed domains while mixed hospitals with ICU scored better in human resources and support services. A similar observation was made by Magluta et al [23] after an investigation of maternity wards in Rio de Janeiro. Those authors concluded that the infrastructure of the hospitals improved as the level of complexity increased.

Indeed, in our study, nearly 40 % of public hospitals without ICU lacked the basic equipment needed for the care of women with obstetric emergencies. This same group of hospitals also had the highest rate of missing medications. Because maternal hemorrhage is one of the leading causes of maternal mortality in Brazil, it is worrying that 43 % of maternity wards without an ICU also lacked blood banks or transfusion units. This is a cause of great concern as Brazil has high rates of caesarian sections, even in low risk women [12], and caesarian sections are associated with increased risk of bleeding complications [24] and need of blood transfusion [25, 26].

The distribution of public and mixed services, according to the availability of ICU, was unequal among the Brazilian macro regions. Public hospitals with ICU were also less available in non-capital cities. Leal and Viacava [27] pointed out this situation in an analysis of Brazilian maternity wards conducted in the year 1999. In that study, ICU facilities were less available in non-capital cities and in less developed regions (North and Northeast). The persistence of this unequal distribution after more than a decade reinforces the need of investments in maternity care in these regions that have the highest burdens of maternal morbidity and mortality [15, 28]. These are also the regions where national studies have uncovered failures of the public system to adequately provide prenatal care and patient transfer to center-of-excellence hospitals, which are often located in the capitals [4, 28, 29].

Studies have suggested that great strides in maternal health can be made by guaranteeing women with obstetric complications access to maternity wards that are qualified to meet their needs [7, 30]. Therefore, it is problematic that, according to our findings, hospitals without ICU or in those with inadequate or partially adequate ICU [8] admitted a high percentage of women at obstetric risk. Previous studies have noted a limited access to hospitals with a higher level of care in the city of Belo Horizonte [31], in the state of Paraná [32] and in the public hospital system [33].

We identified a high proportion of women classified as low obstetric risk admitted to labor and childbirth care in hospitals with ICU. This was more frequent in hospitals located in the Southeast/South/Central-west regions and in capitals, reaching 70.6 % among mixed hospitals located in capital cities. This type of allocation represents unnecessary spending of resources and potential exposure of low-risk pregnant women to unneeded interventions [34]. Results from the Birth in Brazil study regarding obstetric interventions carried out in Brazilian maternity wards show that pregnant women are subjected to routine procedures that do not take into account their individual needs [35]. A study carried out in the United Kingdom compared care setting to maternal and neonatal outcomes in low-risk women; those who gave birth at freestanding midwifery units or alongside midwifery units were not at greater risk for complications, lending support to the lower use of obstetric interventions for pregnant women attended by these types of services [36].

## Conclusion

The overview presented in this study brings up issues relevant to the debate about the quality of hospital services for pregnant women in Brazil. Our results support the view that in many maternity wards across the country, labor and delivery care is unacceptable. This holds true for women at high obstetric risk, who suffer the possibility of having their care compromised by failures of hospital infrastructure, and for women at low obstetric risk, who may not receive the appropriate care to support the natural evolution of their labor when in a technological hospital environment. Changes are needed in hospital structure, as well as in implementation of regional networks to increase access, promote equity and integration of services, and continually improve maternal morbidity and mortality outcomes. The results of this study support the creation of management and policy strategies towards these aims, and argue that further action is indispensable. Future studies are needed to evaluate the care infrastructure of private hospitals and the weight of different items of the adequacy assessment according to different needs of maternal care.
